# Long COVID and the development of new-onset uveitis: a large database study

**DOI:** 10.1186/s12348-025-00527-0

**Published:** 2025-11-04

**Authors:** Qais A. Dihan, Nayef Alshammari, Abdelrahman M. Elhusseiny, Kaersti L. Rickels, Ahmed F. Shakarchi, Muhammad Z. Chauhan, Ahmed B. Sallam

**Affiliations:** 1 Chicago Medical School, Rosalind Franklin University of Medicine and Science, North Chicago, Illinois USA; 2 Department of Ophthalmology, Harvey and Bernice Jones Eye Institute, University of Arkansas for Medical Sciences, 4301 W. Markham Street, Little Rock, AR 72207 USA; 3 Department of Ophthalmology, Harvard Medical School, Boston Children’s Hospital, Boston, MA USA; 4 Department of Ophthalmology, Faculty of Medicine, Ain Shams University, Cairo, Egypt

**Keywords:** Uveitis, COVID, COVID vaccination, Long COVID, Anterior uveitis

## Abstract

**Purpose:**

To determine the impact of long COVID diagnosis on the risk of developing uveitis among individuals vaccinated and not vaccinated against COVID.

**Methods:**

We conducted a population-based retrospective cohort study using an aggregate healthcare database, TriNetX, which includes data from over 127 million patients across 95 international healthcare organizations. Four cohorts were compared: (1) Unvaccinated, Long COVID; (2) Unvaccinated, No Long COVID; (3) Vaccinated, Long COVID; and (4) Vaccinated, No Long COVID. Patients with any history of uveitis prior to initial COVID diagnosis were excluded. The primary outcome was the risk of new-onset uveitis at 1 and 2 years following the diagnosis of long COVID.

**Results:**

Unvaccinated, long COVID patients demonstrated an increased risk of developing new-onset uveitis compared to unvaccinated, no long COVID controls at 1 year (aHR: 2.01, 95% CI: 1.19–3.38) and 2 years (aHR: 1.60, 95% CI: 1.08–2.37). The highest risk was seen for anterior uveitis at 1 year (aHR: 1.96, 95% CI: 1.13–3.41) and 2 years (aHR: 1.59, 95% CI: 1.06–2.40). Other uveitis subtypes did not show an increased risk in this cohort. Among vaccinated individuals, there was not increased risk in those with long COVID compared to those without at 1 year (aHR: 0.95, 95% CI: 0.58–1.55) and 2 years (aHR: 0.97, 95% CI: 0.65–1.46).

**Conclusion:**

Unvaccinated individuals with long COVID have an increased risk of developing new uveitis, particularly anterior uveitis. Vaccinated individuals with long COVID did not have an increased risk of developing uveitis compared to vaccinated non-long COVID individuals.

**Supplementary Information:**

The online version contains supplementary material available at 10.1186/s12348-025-00527-0.

## Introduction

Uveitis refers to the pathological inflammation of the uveal tract, consisting of the iris, ciliary body and choroid [1]. The etiology of uveitis is often not clear, with estimates ranging from 30%−89% of new uveitis diagnoses being classified as idiopathic/undifferentiated [2–4]. Nonetheless, of its known associations, a common thread in affected patients is an underlying, persistent dysregulation of the immune system. This can be noted by the marked association of uveitis with HLA-B27-associated systemic diagnoses, uveitis following the reactivation of latent infections, and uveitis following abrupt CD4 + cell count recovery [5–8]. Currently, acute COVID-19 infection has been implicated as a risk factor for the development of both new-onset and recurrent bouts of uveitis [9].

Long COVID (also termed “post-acute sequelae of COVID-19”) is defined by the World Health Organization as the continuation or development of new symptoms three months following an initial SARS-CoV-2 diagnosis, with symptoms lasting for at least 2 months [10]. Long COVID has been associated with long-term perturbations of the immune system including reduced CD4 + and CD8 + effector T-cell counts, and abnormal long-term elevations in pro-inflammatory mediators [11–13].

The role of COVID-19 vaccination in developing new or recurrent uveitis has been analyzed. Several reports have posited associations between COVID-19 vaccination and the development of new or recurrent uveitis [14–18]. However, a recent report by Kim et al. found no increased risk of uveitis in vaccine recipients [19]. Thus, conflicting reports make it difficult to characterize the exact role of COVID-19 vaccination in the subsequent development of uveitis.

Given the underlying immune dysregulation associated with long COVID, we sought to analyze the association between long COVID and the development of new-onset uveitis. Furthermore, we also sought to separately characterize this relationship amongst vaccinated individuals and unvaccinated individuals.

## Methods

### Standard protocol approvals and principles

The University of Arkansas for Medical Sciences Institutional Review Board exempted this study due to the de-identified nature of the collected data. This study adhered to the principles of the Declaration of Helinski (version 2013).

### Study design

We conducted a population-based retrospective cohort study using TriNetX, an aggregate healthcare database of over 127 million deidentified patients spanning across 95 healthcare institutions. We accessed data from the TriNetX’s COVID-19 Research network on May 17, 2024. To conduct our research queries, we employed codes from the International Classification of Diseases, 10th Revision (ICD-10).

### Participants, cohorts, and propensity score matching

We identified all patients 18 years and older who had undergone COVID-19 testing from between 03/01/2021 to 03/01/2023. COVID-19 diagnosis was confirmed via positive ribonucleic acid (RNA) COVID-19 test result or confirmed clinical diagnosis of COVID-19 (ICD-10: U07.1). Amongst these COVID-19 positive patients, we identified those who went on to develop long COVID using a published approach by the FDA Sentinel Initiative on strategy for the identification of patients with long COVID on TriNetX (ICD-10: B94.8 or U09.9) [20]. Furthermore, we also identified if patients had ever received a vaccination against COVID-19. Amongst our vaccinated study population, patients were only designated as “vaccinated” if they received at least one dose a minimum of one month before their diagnosis of COVID-19, to allow a buffer time in recipients for vaccines to take effect. We excluded patients who received unspecified COVID vaccinations (CVX: 213).

**Fig. 1:**
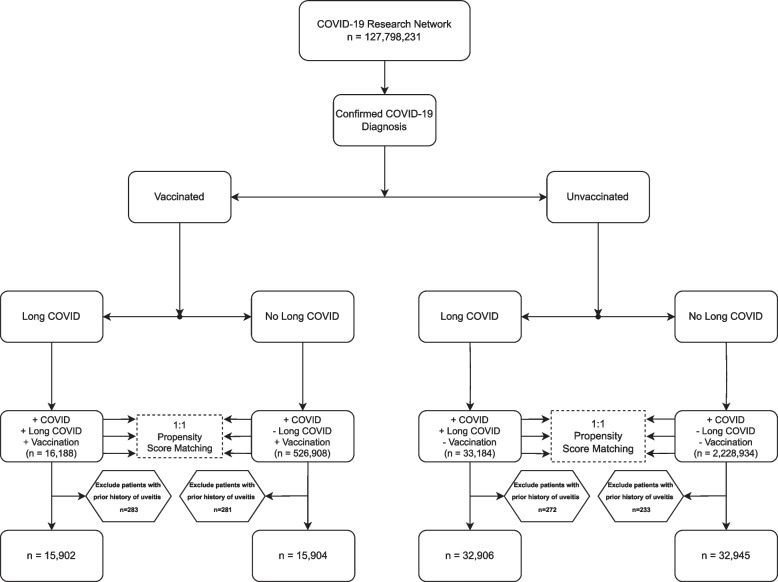
Cohort design, characteristics, and comparisons of COVID-19 positive, patients stratified by long COVID diagnosis and vaccination status

We divided our study participants into 4 cohorts — all of whom shared a common history of at least a one-time confirmed COVID-19 diagnosis. The four cohorts were as follows: never vaccinated patients who never went on to develop long COVID, vaccinated patients who never went on to develop long COVID, never vaccinated patients with a confirmed subsequent diagnosis of long COVID, and vaccinated patients with confirmed subsequent diagnosis of long COVID (Fig. 1). For all patients, the index date was defined as the day on which they met their selected cohort’s inclusion criteria.

We utilized 1:1 propensity score matching via TriNetX’s platform to match cohorts on baseline characteristics and potential risk factors for the development of uveitis. We matched cohorts for variables including age, sex, race, ethnicity, tobacco use, obesity, diabetes mellitus, systemic connective tissue disorders, and coexisting autoimmune conditions including ankylosing spondylitis, rheumatoid arthritis, sarcoidosis, Reiter’s disease, relapsing polychondritis, and others. In all cohorts, we excluded patients with a prior history of uveitis (Tables 1 and 2). We stratified uveitis anatomically into anterior, intermediate, posterior, or pan-uveitis. E-Supplement 1 details the specific ICD-10 codes corresponding to each outcome analyzed, including each subtype of uveitis.

**Table 1:** Unvaccinated patient cohort: before and after propensity score matching

	**Before Matching**				**After Matching**			
**Baseline Patient Characteristics**	Unvaccinated, Long COVID (*n* = 33,184)	Unvaccinated, no Long COVID (*n* = 2,228,934)	Std Diff	*P*-Value	Unvaccinated, Long COVID (*n* = 33,178)	Unvaccinated, no Long COVID (*n* = 33,178)	Std. Diff	*P*-Value
Age, mean (SD)	54.5 (16.8)	48.5 (19.5)	0.329	< 0.001	54.5 (16.8)	54.5 (16.9)	0.002	0.801
Sex, No. (%)								
Female	20,459 (61.6)	1,244,283 (55.8)	0.119	< 0.001	20,457 (61.7)	20,464 (61.7)	< 0.001	0.955
Male	10,740 (32.4)	912,895 (41.0)	0.179	< 0.001	10,739 (32.4)	10,738 (32.4)	< 0.001	0.993
Not Reported	1985 (6.0)	71,756 (3.2)	0.132	< 0.001	1982 (6.0)	1976 (6.0)	0.001	0.922
Race, No. (%)								
White	22,783 (68.7)	1,316,469 (59.1)	0.201	< 0.001	22,780 (68.7)	22,911 (69.1)	0.009	0.272
Black or African American	3461 (10.4)	321,367 (14.4)	0.121	< 0.001	3461 (10.4)	3423 (10.3)	0.004	0.629
Asian	1022 (3.1)	63,787 (2.9)	0.013	0.018	1022 (3.1)	1007 (3.0)	0.003	0.735
Native Hawaiian or Other Pacific Islander	289 (0.9)	14,012 (0.6)	0.028	< 0.001	289 (0.9)	282 (0.9)	0.002	0.769
American Indian or Alaska Native	162 (0.5)	8377 (0.4)	0.017	< 0.001	162 (0.5)	163 (0.5)	< 0.001	0.956
Other Race	1170 (3.5)	97,928 (4.4)	0.045	< 0.001	1170 (3.5)	1136 (3.4)	0.006	0.471
Unknown Race	4297 (13.0)	406,994 (18.3)	0.147	< 0.001	4294 (12.9)	4256 (12.8)	0.003	0.660
Ethnicity, No. (%)								
Hispanic or Latino	2297 (6.9)	182,724 (8.2)	0.048	< 0.001	2297 (6.9)	2255 (6.8)	0.005	0.519
Not Hispanic or Latino	24,863 (74.9)	1,377,741 (61.8)	0.285	< 0.001	24,860 (74.9)	24,962 (75.2)	0.007	0.360
Unknown Ethnicity	6024 (18.2)	668,469 (30.0)	0.280	< 0.001	6021 (18.2)	5961 (17.8)	0.005	0.545
**Comorbidities and Risk Factors**	Unvaccinated, Long COVID (*n* = 33,184)	Unvaccinated, no Long COVID (*n* = 2,228,934)	Std Diff	*P*-Value	Unvaccinated, Long COVID (*n* = 33,178)	Unvaccinated, no Long COVID (*n* = 33,178)	Std. Diff	*P*-Value
Overweight and obesity [E66]	13,009 (39.2)	369,862 (16.6)	0.521	< 0.001	13,003 (39.2)	13,111 (39.5)	0.007	0.391
Diabetes mellitus [E08-E13]	8902 (26.8)	293,058 (13.2)	0.347	< 0.001	8898 (26.8)	9004 (27.1)	0.007	0.354
Tobacco use [Z72.0]	3606 (10.9)	129,322 (5.8)	0.184	< 0.001	3603 (10.9)	3708 (11.2)	0.010	0.193
Other disorders of cartilage [M94]	2847 (8.6)	72,930 (3.3)	0.226	< 0.001	2844 (8.6)	2834 (8.5)	0.001	0.890
Systemic connective tissue disorders [M30-36]	2743 (8.3)	58,693 (2.6)	0.250	< 0.001	2737 (8.3)	2678 (8.1)	0.006	0.403
Other rheumatoid arthritis [M06]	1675 (5.1)	37,944 (1.7)	0.186	< 0.001	1670 (5.0)	1521 (4.6)	0.021	0.007
Other thrombophilia [D68.6]	594 (1.8)	9091 (0.4)	0.133	< 0.001	591 (1.8)	531 (1.6)	0.014	0.071
Unspecified inflammatory spondylopathy [M46.9]	543 (1.6)	10,881 (0.5)	0.112	< 0.001	539 (1.6)	501 (1.5)	0.009	0.235
Rheumatoid arthritis with rheumatoid factor [M05]	460 (1.4)	9526 (0.4)	0.101	< 0.001	458 (1.4)	377 (1.1)	0.022	0.005
Sarcoidosis [D86]	277 (0.8)	6785 (0.3)	0.071	< 0.001	277 (0.8)	253 (0.8)	0.008	0.295
Ankylosing spondylitis [M45]	186 (0.6)	3558 (0.2)	0.067	< 0.001	182 (0.6)	153 (0.5)	0.012	0.112
Juvenile arthritis [M08]	81 (0.2)	1530 (0.1)	0.044	< 0.001	77 (0.2)	60 (0.2)	0.011	0.146
Reiter’s disease [M02.3]	50 (0.2)	705 (0.0)	0.039	< 0.001	49 (0.2)	34 (0.1)	0.013	0.099
Enteropathic arthropathies [M07]	24 (0.1)	452 (0.0)	0.024	< 0.001	24 (0.1)	19 (0.1)	0.006	0.446
Other reactive arthropathies [M02.8]	10 (0.0)	54 (0.0)	0.022	< 0.001	10 (0.0)	0 (0.0)	0.025	0.002
Reactive arthropathy, unspecified [M02.9]	10 (0.0)	171 (0.0)	0.016	< 0.001	10 (0.0)	10 (0.0)	< 0.001	1.000
Relapsing polychondritis [M94.1]	10 (0.0)	150 (0.0)	0.017	< 0.001	10 (0.0)	10 (0.0)	< 0.001	1.000
	**Before Matching**				**After Matching**			
**Uveitis Outcomes (1 year)**	Unvaccinated, Long COVID (*n* = 33,184)	Absolute Risk (%)	Unvaccinated, no Long COVID (*n* = 2,228,934)	Absolute Risk (%)	Unvaccinated, Long COVID (*n* = 33,178)	Absolute Risk (%)	Unvaccinated, Long COVID (*n* = 33,178)	Absolute Risk (%)
**Uveitis (Total)**	42	0.13%	1133	0.05%	42	0.13%	21	0.06%
Anterior Uveitis	37	0.11%	962	0.04%	37	0.11%	19	0.06%
Intermediate Uveitis	≤ 10	0.03%	45	0.002%	≤ 10	0.03%	0	0%
Posterior Uveitis	≤ 10	0.03%	201	0.009%	≤ 10	0.03%	≤ 10	0.03%
Panuveitis	≤ 10	0.03%	40	0.002%	≤ 10	0.03%	0	0%
**Uveitis Outcomes (2 years)**	Unvaccinated, Long COVID (*n* = 33,184)	Absolute Risk (%)	Unvaccinated, no Long COVID (*n* = 2,228,934)	Absolute Risk (%)	Unvaccinated, Long COVID (*n* = 33,178)	Absolute Risk (%)	Unvaccinated, Long COVID (*n* = 33,178)	Absolute Risk (%)
**Uveitis (Total)**	61	0.19%	1877	0.08%	61	0.19%	42	0.13%
Anterior Uveitis	56	0.17%	1634	0.07%	56	0.17%	39	0.12%
Intermediate Uveitis	≤ 10	0.03%	65	0.003%	≤ 10	0.03%	≤ 10	0.03%
Posterior Uveitis	≤ 10	0.03%	297	0.01%	≤ 10	0.03%	≤ 10	0.03%
Panuveitis	≤ 10	0.03%	79	0.004%	≤ 10	0.03%	≤ 10	0.03%

**Table 2:** Vaccinated patient cohort: before and after propensity score matching

	**Before Matching**				**After Matching**			
**Baseline Patient Characteristics**	Vaccinated, Long COVID (*n* = 16,188)	Vaccinated, no Long COVID (*n* = 526,908)	Std Diff	*P*-Value	Vaccinated, Long COVID (*n* = 16,185)	Vaccinated, no Long COVID (*n* = 16,185)	Std. Diff	*P*-Value
Age, mean (SD)	57.2 (16.7)	53.2 (19.1)	0.223	< 0.001	57.2 (16.7)	57.4 (16.8)	0.011	0.302
Sex, No. (%)								
Female	10,541 (65.1)	316,210 (60.0)	0.106	< 0.001	10,538 (65.1)	10,519 (65.0)	0.002	0.825
Male	5127 (31.7)	198,379 (37.7)	0.126	< 0.001	5127 (31.7)	5150 (31.8)	0.003	0.784
Not Reported	621 (3.8)	33,325 (6.3)	0.114	< 0.001	621 (3.8)	595 (3.7)	0.008	0.447
Race, No. (%)								
White	11,533 (71.2)	350,667 (66.6)	0.101	< 0.001	11,530 (71.2)	11,580 (71.5)	0.007	0.539
Black or African American	1206 (7.5)	46,725 (8.9)	0.052	< 0.001	1206 (7.5)	1176 (7.3)	0.007	0.523
Asian	520 (3.2)	12,319 (2.3)	0.053	< 0.001	520 (3.2)	516 (3.2)	0.001	0.899
Native Hawaiian or Other Pacific Islander	94 (0.6)	3182 (0.6)	0.003	0.71	94 (0.6)	109 (0.7)	0.012	0.291
American Indian or Alaska Native	65 (0.4)	1662 (0.3)	0.014	0.06	65 (0.4)	66 (0.4)	0.001	0.930
Other Race	612 (3.8)	23,992 (4.6)	0.039	< 0.001	612 (3.8)	583 (3.6)	0.010	0.393
Not Reported	1545 (9.5)	54,337 (10.3)	0.026	< 0.001	1545 (9.5)	1507 (9.3)	0.008	0.470
Ethnicity, No. (%)								
Hispanic or Latino	1121 (6.9)	47,573 (9.0)	0.078	< 0.001	1121 (6.9)	1067 (6.6)	0.013	0.232
Not Hispanic or Latino	13,522 (83.5)	424,998 (80.7)	0.075	< 0.001	13,519 (83.5)	13,611 (84.1)	0.015	0.165
Not Reported	2057 (12.7)	67,355 (12.8)	0.002	0.78	2057 (12.7)	2076 (12.8)	0.004	0.752
**Comorbidities and Risk Factors**								
Overweight and obesity [E66]	8029 (49.6)	167,103 (31.7)	0.370	< 0.001	8026 (49.6)	8065 (49.8)	0.005	0.665
Diabetes mellitus [E08-E13]	5853 (36.2)	129,878 (24.6)	0.252	< 0.001	5851 (36.2)	5886 (36.4)	0.004	0.686
Tobacco use [Z72.0]	3412 (21.1)	77,772 (14.8)	0.165	< 0.001	3410 (21.1)	3468 (21.4)	0.009	0.431
Other disorders of cartilage [M94]	2364 (14.6)	37,543 (7.1)	0.242	< 0.001	2361 (14.6)	2342 (14.5)	0.003	0.764
Systemic connective tissue disorders [M30-36]	1883 (11.6)	36,483 (6.9)	0.163	< 0.001	1880 (11.6)	1856 (11.5)	0.005	0.676
Other rheumatoid arthritis [M06]	1199 (7.4)	19,747 (3.7)	0.160	< 0.001	1196 (7.4)	1132 (7.0)	0.015	0.169
Other thrombophilia [D68.6]	447 (2.8)	7147 (1.4)	0.099	< 0.001	446 (2.8)	395 (2.4)	0.020	0.075
Unspecified inflammatory spondylopathy [M46.9]	355 (2.2)	5710 (1.1)	0.087	< 0.001	354 (2.2)	325 (2.0)	0.013	0.261
Rheumatoid arthritis with rheumatoid factor [M05]	328 (2.0)	4371 (0.8)	0.101	< 0.001	325 (2.0)	284 (1.8)	0.019	0.093
Sarcoidosis [D86]	224 (1.4)	3602 (0.7)	0.069	< 0.001	223 (1.4)	199 (1.2)	0.013	0.240
Ankylosing spondylitis [M45]	121 (0.8)	1967 (0.4)	0.050	< 0.001	119 (0.7)	90 (0.6)	0.022	0.044
Juvenile arthritis [M08]	38 (0.2)	831 (0.2)	0.017	0.02	38 (0.2)	28 (0.2)	0.014	0.218
Reiter’s disease [M02.3]	27 (0.2)	352 (0.1)	0.029	< 0.001	27 (0.2)	21 (0.1)	0.010	0.386
Enteropathic arthropathies [M07]	23 (0.1)	363 (0.1)	0.023	< 0.001	22 (0.1)	17 (0.1)	0.009	0.423
Other reactive arthropathies [M02.8]	14 (0.1)	145 (0.0)	0.025	< 0.001	13 (0.1)	10 (0.1)	0.007	0.531
Reactive arthropathy, unspecified [M02.9]	10 (0.1)	27 (0.0)	0.031	< 0.001	10 (0.1)	0 (0.0)	0.035	0.002
Relapsing polychondritis [M94.1]	10 (0.1)	103 (0.0)	0.021	< 0.001	10 (0.1)	10 (0.1)	< 0.001	1.000
	**Before Matching**				**After Matching**			
**Uveitis Outcomes (1 year)**	Vaccinated, Long COVID (*n* = 16,188)	Absolute Risk (%)	Vaccinated, no Long COVID (*n* = 526,908)	Absolute Risk (%)	Vaccinated, Long COVID (*n* = 16,185)	Absolute Risk (%)	Vaccinated, no Long COVID (*n* = 16,185)	Absolute Risk (%)
**Uveitis (Total)**	30	0.19%	672	0.13%	30	0.19%	34	0.21%
Anterior Uveitis	30	0.19%	582	0.11%	30	0.19%	32	0.20%
Intermediate Uveitis	≤ 10	0.062%	24	0.005%	0	0%	≤ 10	0.062%
Posterior Uveitis	≤ 10	0.062%	104	0.02%	0	0%	≤ 10	0.062%
Panuveitis	0	0%	29	0.006%	0	0%	≤ 10	0.062%
**Uveitis Outcomes (2 years)**	Vaccinated, Long COVID (*n* = 16,188)	Absolute Risk (%)	Vaccinated, no Long COVID (*n* = 526,908)	Absolute Risk (%)	Vaccinated, Long COVID (*n* = 16,185)	Absolute Risk (%)	Vaccinated, no Long COVID (*n* = 16,185)	Absolute Risk (%)
**Uveitis (Total)**	44	0.28%	1040	0.20%	43	0.27%	52	0.33%
Anterior Uveitis	43	0.27%	902	0.17%	43	0.27%	46	0.29%
Intermediate Uveitis	≤ 10	0.062%	39	0.007%	≤ 10	0.062%	≤ 10	0.062%
Posterior Uveitis	≤ 10	0.062%	171	0.03%	≤ 10	0.062%	≤ 10	0.062%
Panuveitis	0	0%	44	0.008%	0	0%	≤ 10	0.062%

### Outcomes

The primary outcome of the study was the development of new-onset uveitis at 1 and 2 years following initial COVID diagnosis. We also conducted a secondary analysis of the anatomical type of uveitis.

### Statistical analysis

We presented the baseline characteristics as means with standard variations for continuous variables and counts with relative cohort percentages for binary variables. We reported the selected characteristics before and after propensity score matching. Thereafter, we sought to quantify the risk of developing new-onset uveitis between cohorts. We utilized Cox proportional hazard models to calculate adjusted hazard ratios (aHR) and their associated 95% confidence intervals (CI) at 1 and 2-year time points. All statistical tests between cohorts were two-sided, and we set the threshold for statistical significance at *P* <.05.

## Results

### Patient characteristics

The mean age of vaccinated patients was 57 years of age, and the mean age of unvaccinated patients was 54 years of age. Females comprised a modest majority of our unvaccinated cohort (61.7%) and vaccinated cohort (65.0%) after matching (see Tables 1 and 2). The three most common comorbidities identified amongst both vaccinated and unvaccinated long COVID patients were obesity, diabetes mellitus, and tobacco use.

### The role of long COVID on uveitis development in an unvaccinated patient cohort

Our first cohort comparison involved analyzing the rates of new uveitis development in long COVID patients who never received a COVID vaccination. We identified 33,184 unvaccinated patients who developed long COVID following a confirmed COVID diagnosis, and 2,228,934 unvaccinated patients who did not develop long COVID following a confirmed COVID diagnosis. None of these patients had a documented history of uveitis before their initial diagnosis of COVID.

Before propensity score matching, we found the overall risk for developing uveitis in unvaccinated patients with long COVID was 0.13% at 1 year, and 0.18% at 2 years. The overall risk for developing uveitis in unvaccinated patients with no long COVID was 0.05% at 1 year, and 0.08% at 2 years. After propensity score matching, there were 33,178 (long COVID) vs 33,178 (no long COVID) patients in our comparison cohorts (Table 1). Our Cox proportional hazards regression model showed a higher risk of developing any type of uveitis in unvaccinated long COVID patients vs unvaccinated no long COVID patients, at 1 year (aHR 2.01, 95% CI: 1.19–3.38, *P* =.01) and 2 years (aHR: 1.60, 95% CI: 1.08–2.37, *P* =.02).

On secondary analysis, the most common subtype of uveitis developed at 1 and 2 years was anterior uveitis, representing 58.3% and 61.3% of total recorded uveitis diagnoses, respectively. Risk for anterior uveitis was also increased in unvaccinated long COVID patients at 1 year (aHR: 1.96, 95% CI: 1.13–3.41, *P* =.02) and 2 years (aHR: 1.59, 95% CI: 1.06–2.40, *P* =.02). The risk of intermediate, posterior, and pan- uveitides were not elevated at 1 or 2 years.

### The role of long COVID on uveitis development in a vaccinated patient cohort

Our second comparison involved analyzing the rates of new uveitis development in long COVID patients who were vaccinated against COVID-19. We identified 16,188 vaccinated patients who developed long COVID following a confirmed COVID diagnosis, and 526,908 vaccinated patients who did not develop long COVID following a confirmed COVID diagnosis. None of these patients had a documented history of uveitis before their initial COVID diagnosis or vaccination.

Before propensity score matching, we found the overall risk for developing uveitis in vaccinated patients with long COVID was 0.19% at 1 year and 0.28% at 2 years. The overall risk for developing uveitis in vaccinated patients with no long COVID was 0.13% at 1 year, and 0.20% at 2 years. After propensity score matching, there were 16,185 (long COVID) and 16,185 (no long COVID) patients in our comparison cohorts. Table 2 shows cohorts before and after propensity score matching.

Our Cox proportional hazards regression model showed no elevated risk for developing any type of uveitis in vaccinated long COVID patients as compared to no long COVID vaccinated patients at 1 year (aHR: 0.95, 95% CI: 0.58–1.55, *P* =.76) or 2 years (aHR: 0.97, 95% CI: 0.65–1.46, *P* =.88). On subsequent secondary analysis, there was no elevated risk of developing any subtype of uveitis.

## Discussion

This population-based retrospective cohort study examined the role of long COVID in patients developing new-onset uveitis. We found an increased risk of anterior uveitis in long COVID patients compared to patients without long COVID among unvaccinated individuals. There was no increased risk of other types of uveitis. Among vaccinated individuals, there was no significant difference in uveitis risk by long COVID status. Of note, vaccinated individuals had an overall higher rate of uveitis.

Though no studies have analyzed the role of long COVID as a risk factor for developing uveitis, acute COVID-19 infection has been implicated as a risk factor for the development of uveitis. In a case series by Feng et al., subjects demonstrated an increased risk of developing uveitis within one month of COVID-19 infection [9]. In a larger study by Hsia et al., COVID-19 patients demonstrated an increased risk of uveitis up to 24 months after infection, when compared to patients without COVID-19 [21]. Though Hsia and colleagues did not consider long COVID in their analysis, our timeline for increased risk was similar; we noted an increased risk of developing new-onset uveitis up to 24 months after a long COVID diagnosis.

In comparison to COVID-19 infection, studies examining the association between COVID-19 vaccinations and uveitis are more prevalent in the literature. A nationwide retrospective cohort study by Chang et al. noted a modest increased risk in non-anterior uveitis up to 6 months after vaccination, but concluded given the modest effect size they would still recommend vaccination to patients [22]. A more recent 2024 study of nearly 8 million individuals by Kim et al. found no increased risk of uveitis following the first dose of COVID-19 vaccination [19].

Age may also be a related factor, which may contribute to uveitis risk. Yeung et al. demonstrated that uveitis occurred at a greater rate in females who were above the age of 50, citing that females over 50 had a greater predisposition than males to develop an autoimmune condition, which may precipitate the development of uveitis [23]. As demonstrated in Tables 1 and 2, the mean age of uveitis patients in our study was over 50 years of age on average and were female. Thus, our findings mirrored these trends. Amongst our noted patient risk factors, it is also important to mention that the top three most common risk factors amongst long COVID patients were obesity, diabetes mellitus, and tobacco use. All three factors have been documented to increase the risk for development of long COVID [24–26]. In addition to developing long COVID, proinflammatory components in tobacco smoke, and a persistent low-grade inflammation noted in diabetes and obesity may also increase the risk of developing uveitis [27–31].

Our sub analysis demonstrated an increased risk of anterior uveitis in unvaccinated, long COVID patients, but noted no other appreciable risk increase for other subtypes. Anterior uveitis has frequently been implicated in autoimmune conditions, and systemic autoimmune diseases may result in a breakdown of the eye’s anterior chamber-associated immune deviation (ACAID) mechanism, which works to suppress overt inflammation in the anterior chamber and preserve tissue transparency [32]. Thus, given the aforementioned systemic autoimmunity that may occur in long COVID, this may explain why this subtype was most common in long COVID patients in our study [5, 33]. Nevertheless, the connection between long COVID and uveitis is invariably complex, and factors including sex, gender, environment, and other comorbidities may also play a role.

While our study did not demonstrate vaccination to increase the risk of uveitis among long COVID patients, an important consideration established by prior research has demonstrated that patient anxiety and concern regarding vaccination can influence the perception and reporting of symptoms, potentially amplifying subjective symptom severity without objective disease progression [34]. This underscores the importance of physicians addressing patient concerns and providing psychological reassurance when discussing vaccination. Alleviating anxiety not only enhances patient mental well-being but may also mitigate the perception of physical symptoms, fostering greater confidence in vaccination and adherence to care plans.

Our study has several limitations. First, our study does not establish cause and effect. Rather we analyzed the rates of occurrence between co-occurring conditions and sought to establish an association between long COVID and uveitis. Further studies are warranted on a direct causal link between long COVID and uveitis. Second, differences in baseline risk factors may have existed between our cohorts that were not able to be accounted for between cohorts. We did seek to mitigate these differences via TriNetX’s analytics platform to match cohorts via propensity score matching, However, our study involved the use of separate propensity score matching for each cohort, which confines the applicability of comparative risks to intra-cohort analyses (by vaccination status). While this method allows for robust comparisons within each cohort (e.g., unvaccinated patients with long COVID vs. unvaccinated patients without long COVID), it precludes the ability to draw conclusions between cohorts (e.g., unvaccinated vs. vaccinated patients). This is because each cohort’s propensity score matching is tailored to its own set of baseline characteristics and risk factors, rendering inter-cohort comparisons non-applicable. Third, our study made use of specified diagnosis codes to identify patients with defined outcomes, yet at times diagnosis codes may be limited as they may confine patients to particular codes that may not fully describe the scope of their condition. This is true with relation to the definition of long COVID and its associated ICD-10 codes – thus we utilized an analytical approach from a published report on how to best capture long COVID patients from the international database we utilized [20]. Furthermore, our study analyzed nearly 2.9 million patient records, and thus our large sample size may have provided value in reducing potential ICD-10 reporting errors or inaccuracies.

In conclusion, our population-based retrospective cohort study demonstrated an increased risk of the development of new uveitis in unvaccinated, long COVID patients. An increased risk of anterior uveitis, in particular, was noted in this cohort. Amongst vaccinated patients, no increased risk of developing new uveitis was noted, regardless of if they developed long COVID. COVID-19 vaccination appeared to be protective against new uveitis in this cohort.

## Supplementary Information

Supplementary Material 1: E-Supplement 1. Diagnosis codes utilized to define outcomes, and comorbid/risk factors used for propensity score matching for confirmed COVID-19 positive patients, stratified by long COVID diagnosis and vaccination status.

## Data Availability

No datasets were generated or analysed during the current study.
