# Supplementary material for: Long COVID and the development of new-onset uveitis: a large database study
Source: J Ophthalmic Inflamm Infect. 2025 Nov 4;15:79. doi: 10.1186/s12348-025-00527-0 (PMC12586249; doi:10.1186/s12348-025-00527-0)
Supplement: Supplementary file 1 — Supplementary Material 1: E-Supplement 1. Diagnosis codes utilized to define outcomes, and comorbid/risk factors used for propensity score matching for confirmed COVID-19 positive patients, stratified by long COVID diagnosis and vaccination status. [file 12348_2025_527_MOESM1_ESM.docx]

E-Supplement 1: Diagnosis Codes Used for Cohort Setup in TriNetX Analysis

| **COVID Infection** |  |  |  |
| --- | --- | --- | --- |
|  | **Type** | **Code** | **Description** |
|  | LOINC | 94309-2 | SARS-CoV-2 (COVID-19) RNA [Presence] in Specimen by NAA with probe detection [Result: **Positive**] |
|  | LOINC | 94316-7 | SARS-CoV-2 (COVID-19) N gene [Presence] in Specimen by NAA with probe detection [Result: **Positive**] |
|  | LOINC | 94500-6 | SARS-CoV-2 (COVID-19) RNA [Presence] in Respiratory system Specimen by NAA with probe detection [Result: **Positive**] |
|  | LOINC | 94533-7 | SARS-CoV-2 (COVID-19) N gene [Presence] in Respiratory system Specimen by NAA with probe detection [Result: **Positive**] |
|  | LOINC | 94559-2 | SARS-CoV-2 (COVID-19) ORF1ab region [Presence] in Respiratory system Specimen by NAA with probe detection [Result: **Positive**] |
|  | LOINC | 94565-9 | SARS-CoV-2 (COVID-19) RNA [Presence] in Nasopharynx with non-probe detection [Result: **Positive**] |
|  | TNX-Curated | 9088 | SARS coronavirus 2 and related RNA [Presence] [Result: **Positive**] |
|  | ICD-10 | U07.1 | COVID-19 |
| **Long COVID** | **Type** | **Code** | **Description** |
|  | ICD-10 | B94.9 | Sequelae of other specified infectious and parasitic diseases |
|  | ICD-10 | U09.9 | Post COVID-19 condition, unspecified |
| **COVID Vaccinations** | **Type** | **Code** | **Description** |
|  | CPT | 91300 | Severe acute respiratory syndrome coronavirus 2 (SARS-CoV-2) (coronavirus disease [COVID-19)) vaccine, mRNA-LNP, spike protein, preservative free, 30 mcg/0.3 mL dosage, diluent reconstituted, for intramuscular use |
|  | CPT | 91301 | Severe acute respiratory syndrome coronavirus 2 (SARS-CoV-2) (coronavirus disease [COVID-19]) vaccine, mRNA-LNP, spike protein,  preservative free, 100 mcg/0.5 mL dosage, for intramuscular use |
|  | CPT | 91302 | Severe acute respiratory syndrome coronavirus 2 (SARS-CoV-2) (coronavirus disease [COVID-19]) vaccine, DNA, spike protein, chimpanzee adenovirus Oxford 1 (ChAdOx1) vector, preservative free, 5x1010 viral particles/0.5 mL dosage, for intramuscular use |
|  | CPT | 91303 | Severe acute respiratory syndrome coronavirus 2 (SARS-CoV-2) (coronavirus disease [COVID-19]) vaccine, DNA, spike protein, adenovirus type 26 (Ad26) vector, preservative free, 5x1010 viral particles/0.5 mL dosage, for intramuscular use |
|  | CPT | 91305 | Severe acute respiratory syndrome coronavirus 2 (SARS-CoV-2) (coronavirus disease (COVID-19]) vaccine, mRNA-LNP, spike protein, preservative free, 30 mcg/0.3 mL dosage, tris-sucrose formulation, for intramuscular use |
|  | CPT | 91312 | Severe acute respiratory syndrome coronavirus 2 (SARS-CoV-2) (coronavirus disease [COVID-19)) vaccine, mRNA-274,678 LNP, bivalent spike protein, preservative free, 30 mcg/0.3 mL dosage, tris-sucrose formulation, for intramuscular use |
|  | RxNorm | 2610328 | SARS-CoV-2 (COVID-19) vaccine, mRNA-1273 0.05 MG/ML / SARS-CoV-2 (COVID-19) vaccine, mRNA-1273 OMICRON (BA.4/BA.5) 0.05 MG/ML Injectable Suspension |
|  | RxNorm | 2618347 | 0.3 ML SARS-CoV-2 (COVID-19) vaccine, mRNA-BNT162b2 0.05 MG/ML / SARS-CoV-2 (COVID-19) vaccine, mRNA-BNT16262 OMICRON (BA.4/BA.5) - 1 MG/ML |
|  | CPT | 0001A | Immunization administration by intramuscular injection of severe acute respiratory syndrome coronavirus 2 (SARS-CoV-2) (coronavirus disease (COVID-19)) vaccine, mRNA-LNP, spike protein, preservative free, 30 mcg/0.3 mL dosage, diluent reconstituted; first dose |
|  | CPT | 0011A | Immunization administration by intramuscular injection of severe acute respiratory syndrome coronavirus 2 (SARS-CoV-2) (coronavirus disease [COVID-19]) vaccine, mRNA-LNP, spike protein, preservative free, 100 mcg/0.5 mL dosage; first dose |
|  | CPT | 0021A | Immunization administration by intramuscular injection of severe acute respiratory syndrome coronavirus 2 (SARS-12 CoV-2) (coronavirus disease [COVID-19]) vaccine, DNA, spike protein, chimpanzee adenovirus Oxford 1 (ChAdOx1) vector, preservative free, 5x1010 viral particles/0.5 mL dosage; first dose |
|  | CPT | 0031A | Immunization administration by intramuscular injection of severe acute respiratory syndrome coronavirus 2 (SARS-CoV-2) (coronavirus disease [COVID-19]) vaccine, DNA, spike protein, adenovirus type 26 (Ad26) vector, preservative free, 5x1010 viral particles/0.5 mL dosage; single dose |
|  | CVX | 213 | SARS-CoV-2 (COVID-19) Vaccine - Type Unspecified |
| **Uveitis** | **Type** | **Code** | **Description** |
| Anterior | ICD-10 | H20.0x | Acute and subacute iridocyclitis |
| Anterior | ICD-10 | H20.1x | Chronic iridocyclitis |
| Anterior | ICD-10 | H20.2x | Lens-induced iridocyclitis |
| Anterior | ICD-10 | H20.8x | Other iridocyclitis |
| Anterior | ICD-10 | H20.9x | Iridocyclitis, unspecified |
| Intermediate | ICD-10 | H30.2 | Posterior Cyclitis |
| Posterior | ICD-10 | H30.0x | Focal chorioretinal inflammation |
| Posterior | ICD-10 | H30.1 | Disseminated chorioretinal inflammation |
| Posterior | ICD-10 | H30.8 | Other chorioretinal inflammations |
| Posterior | ICD-10 | H30.9 | Unspecified chorioretinal inflammation |
| Posterior | ICD-10 | H32 | Chorioretinal disorders in diseases classified elsewhere |
| Posterior | ICD-10 | H35.06 | Retinal vasculitis |
| Panuveitis | ICD-10 | H44.11 | Panuveitis |
| **Comorbidities/Risk Factors** | **Type** | **Code** | **Description** |
|  | ICD-10 | E66 | Overweight and obesity |
|  | ICD-10 | E08-E13 | Diabetes mellitus |
|  | ICD-10 | Z72.0 | Tobacco use |
|  | ICD-10 | M94 | Other disorders of cartilage |
|  | ICD-10 | M30-M36 | Systemic connective tissue disorders |
|  | ICD-10 | M06 | Other rheumatoid arthritis |
|  | ICD-10 | D68.6 | Other thrombophilia |
|  | ICD-10 | M46.9 | Unspecified inflammatory spondylopathy |
|  | ICD-10 | M05 | Rheumatoid arthritis with rheumatoid factor |
|  | ICD-10 | D86 | Sarcoidosis |
|  | ICD-10 | M45 | Ankylosing spondylitis |
|  | ICD-10 | M08 | Juvenile arthritis |
|  | ICD-10 | M02.3 | Reiter's disease |
|  | ICD-10 | M07 | Enteropathic arthropathies |
|  | ICD-10 | M02.8 | Other reactive arthropathies |
|  | ICD-10 | M02.9 | Reactive arthropathy, unspecified |
|  | ICD-10 | M94.1 | Relapsing polychondritis |
